# A national CASC course

**DOI:** 10.1192/bjo.2021.427

**Published:** 2021-06-18

**Authors:** Yathooshan Ramesh, Lisanne Stock

**Affiliations:** Barnet, Enfield and Haringey NHS Mental Health Trust

## Abstract

**Aims:**

There was understandable anxiety from trainees about the transition to the online format of the CASC due to the pandemic. There is also significant variability between trusts in the availability of lectures tailored specifically to the CASC exam. Having recent experience of the CASC exam, including the online format, we developed a free online lecture series. We aimed to address common questions relating to the exam, and selected topics that trainees may find daunting or had less experience with through clinical care. The topics covered were: An Introduction to the CASC, Mental State Examinations, Psychological Therapies, Pharmacology and a Q&A Session.

**Method:**

The course was designed to tackle areas that trainees often find difficult based on our own experiences as well as surveying course attendees. Prior to the course, we liaised with consultant site tutors & junior doctor representatives to integrate the course into the local academic programme, and to facilitate promotion of the session to trainees across sites. We subsequently offered registration to trainees nationally. The course was planned and delivered by the organisers through interactive lecture-based presentations with handouts, ahead of the January 2021 examinations. Content was based on national guidelines and published research. 5 sessions were delivered with the final session including guest consultant panellists to answer trainee questions. Quantitative and qualitative feedback was collected from the attendees.

**Result:**

172 doctors registered onto the course, with 44 NHS trusts represented. Doctors from a variety of grades attended, with 55% CT3s, 17% Specialty Doctors, 16% CT2s, 8% CT1s, 4% in other roles. 100% of attendees stated that they would recommend the course to any doctor sitting the CASC. 97% of attendees rated the course as either ‘Excellent’ or ‘Good’. Qualitative feedback was positive and 3 themes were identified- communication, content and the online format.

**Conclusion:**

The CASC course provided an opportunity to deliver national teaching to trainees based on national guidelines and peer-reviewed research, with a focus on addressing areas that trainees may feel less confident with. The course received significant positive feedback from attendees. The significant number of pre-CT3 trainees attending the course suggests that there may be an interest from this group for further support in developing the complex communication skills that ultimately are assessed by the CASC exam.

